# Evaluation of efficacy and efficiency of a pragmatic intervention by a social worker to support informal caregivers of elderly patients (The ICE Study): study protocol for a randomized controlled trial

**DOI:** 10.1186/s13063-016-1622-8

**Published:** 2016-11-03

**Authors:** Astrid Pozet, Catherine Lejeune, Magalie Bonnet, Sandrine Dabakuyo, Michèle Dion, Philippe Fagnoni, Maryse Gaimard, Geneviève Imbert, Virginie Nerich, Audrey Foubert, Morgane Chotard, Marie Bonin, Amélie Anota, Franck Bonnetain

**Affiliations:** 1 Methodology and Quality of Life in Oncology Unit, University Hospital of Besançon, Institut National de la Santé et de la Recherche Médicale UMR 1098, Besançon, France; 2 INSERM U866 Epidémiologie et Recherche Clinique en Oncologie Digestive, University of Burgundy, Dijon, France; 3 UFR Sciences du Langage de l’Homme et de la Société, University of Franche-Comté, Besançon, France; 4 EA 4184, Centre Georges François Leclerc, Dijon, France; 5 Centre Georges Chevrier, UMR CNRS 7366, UFR Sciences Humaines et Sociales, Dijon, France; 6 EA 4184, Faculty of Pharmacy, University of Burgundy, Dijon, France; 7 INSERM U1098. Interaction Hôte-Greffon et Ingénierie Cellulaire et Génique, Besançon, France; 8 Pôle de Gérontologie Interrégional Bourgogne et Franche-Comté (PGI), Besançon, France; 9 The French National Platform Quality of Life and Cancer, Besançon, France; 10 Methodology and Quality of Life in Oncology Unit, University Hospital Jean Minjoz, Boulevard Fleming, 25030 Besançon, France

**Keywords:** Informal caregivers, Elderly, Health-related quality of life, Cost-utility, Randomized study, Longitudinal cohort study, Cancer, Chronic disease

## Abstract

**Background:**

Medical progress and the lifestyle modification have prolonged life expectancy, despite the development of chronic diseases. Support and care for older subjects are often provided by a network of informal caregivers composed of family, friends and neighbors, who are essential in helping older persons to continue living at home. It has been shown that the extent and diversity of informal tasks may jeopardize the physical, mental and social wellbeing of caregivers.

**Methods/design:**

The aim of the Informal Carers of Elderly cohort is to define, through a longitudinal study, profiles of caregivers of older patients with a diagnosis of one of the following diseases: cancer (breast, prostate, colorectal), neurodegenerative diseases (Parkinson’s disease, Alzheimer’s disease and similar diseases), neurovascular diseases (stroke), sensory diseases (age-related macular degeneration (AMD)) and heart disease (heart failure). Patients must be at least 60 years old and living in the region of Burgundy-Franche-Comte (France). By following the different phases of the caregiving relationship from the announcement of the diagnosis, it will be possible to assess the quality of life of caregivers, coping strategies, levels of anxiety and depression, social support and the extent of their burden. We will also evaluate the efficacy and efficiency of the implementation of a pragmatic intervention by a social worker to help informal caregivers, through a randomized interventional trial nested in the cohort. Qualitative approaches aimed at studying the caregiver/patient relationship, and situations leading to breakdown of the caregiver relationship will be also undertaken.

**Discussion:**

Through an analytical and longitudinal definition of profiles of informal caregivers, this study will gather detailed information on their life courses and their health trajectory by identifying consequences associated with the concept of their role as carers. In addition, the randomized interventional trial will explore the relevance of the implementation of a supportive intervention by a social worker to help caregivers. These data will help to identify strategies that could be used to improve the existing sources of aid and to propose new approaches to help caregivers. This study will provide the opportunity to identify the most relevant means of support adapted to caregivers, and provide an impulse for new health care policies.

**Trial registration:**

ClinicalTrials.gov Identifier: NCT02626377. Retrospectively registered on 9 December 2015. Protocol date/version: 23 October 2014/version 2.

## Background

In France, almost four million people provide informal care on a daily basis to people aged 60 years and over [1]. The topic of informal caregiving has become a key issue with regard to care and support for, and consequently maintenance at home of, chronically ill older people, and the features and progress of their dependence. Caregivers play an essential role in providing support to patients [1–4]. Different reasons lead people to become caregivers of a patient, such as chronic disease (51 %), older age (44 %), physical handicap (30 %) and mental handicap (13 %) [2]. Among the various diseases, Alzheimer’s disease is the most frequent cause of becoming an informal caregiver (17 %), followed by age-related macular degeneration (AMD) (15 %), heart failure (14 %), hypertension (13 %), cancer (12 %), stroke (10 %), diabetes (9 %), osteoporosis (8 %) and Parkinson’s disease (4 %) [2]. Over the last few years, informal caregivers have been classified as a frail population with an increased risk of health problems themselves. Psychological components of the informal caregiver role, especially chronic stress, are associated with an increased risk of depression, mortality and cardiovascular or neurovascular morbidity [5–7]. The mean duration of informal caregiving situations is about 7 years, and the delivering of care often has deleterious effects on the caregiver’s general health. Forty percent of caregivers believe themselves to be in poor health, although 90 % of caregivers say that they can cope with the difficulties arising from the care relationship. However, they report a deterioration of their family life (35 %), marital life (41 %), mood (49 %), physical condition (57 %) and leisure activities (70 %) [4].

The psychological distress felt by informal caregivers seems to be directly related to the care provided: the time devoted to care and support, the nature of the care, and behavioral disorders. Such distress could be generated by the effects of personal investment in the role of caregiver, namely a reduction in personal leisure time, loss of social links, family conflict and financial difficulties. The care relationship leads to an increase in workload, which is likely to accentuate any latent health problem of the caregiver [8]. The “adjustment” must be made at the cost of time devoted to family, friends or work. Accordingly, 29 % of caregivers think that there is a lack of moral and emotional support [2]. The difficulties experienced by caregivers may, therefore, have a direct impact on the quality of the care provided to patients [9]. It thus seems essential to identify caregivers’ profiles through a longitudinal follow-up of their life course, to study the course of their quality of life, to understand the context in which care is provided, to identify factors that amplify or attenuate “burn-out,” to measure the impact of support on the caregiver’s life, to understand strategies for coping with the stress, and to identify psychological issues. Another important issue is the identification of the most appropriate type of support that could prevent the deterioration of the caregivers’ physical and mental health, and preserve their social environment, thereby ensuring the continuity of therapeutic care to patients. Various forms of support for caregivers are currently available. Previous studies have underlined the efficacy of these interventions for both caregivers and patients [10]. The effects varied according to the age and gender of the caregiver, the social link between caregivers and patients, the duration, the frequency of the “work/care” and the type of intervention. The patient’s disease is also a key component.

To date, few longitudinal studies of caregivers have been conducted. As a result, there is a lack of data to identify caregivers’ profiles in a dynamic construct perspective. Thus, there is a clear need for scientifically validated knowledge of this population. This is a key step in identifying interventional strategies that could be applied and tailored to the caregivers’ profiles. Our hypothesis is that caregivers represent a heterogeneous group of individuals with different needs and different coping strategies. As suggested by previous reports, the support proposed to informal caregivers should be varied and adapted to the individual. In this respect, the Eurofamcare study underlined the need for a systematic evaluation of the role and needs of caregivers [11]. In addition, a meta-analysis showed the importance of screening for burn-out in caregivers on a 6-monthly basis [12]. Other studies have shown how the circumstances in which a person comes to take on the role of caregiver can affect the caregiver-patient relationship. Indeed, in the long term, the relationship devoted to care may be compromised. In this regard, proactive interventions initiated from the earliest stages of the caregiving relationship seem essential in promoting optimized coping [13–18].

These points led us to undertake a longitudinal cohort study to follow the informal main caregivers of patients who are in the early phase of a diagnosis of breast, prostate or colorectal cancer, Alzheimer’s or similar diseases, Parkinson’s disease, stroke, AMD or heart failure.

## Methods/design

### Trial design

This research project is a prospective, multicenter, longitudinal cohort study incorporating two types of study, namely an observational study and a nested randomized interventional trial among caregivers included in the observational study.

#### Observational study

The primary objective of the observational study is to evaluate, using quantitative approaches, the patterns of informal caregivers for patients aged 60 years and older according to the evaluation of their health-related quality of life (HRQoL) (as assessed by the Medical Outcome Study Short Form-36 items (MOS SF-36) and the CareGiver Oncology Quality of Life (CarGOQoL) questionnaires (only for caregivers of the group of cancer patients), coping strategies (as assessed by the coping scale developed by Borteyrou, Truchot and Rascle [35]), anxiety and depression (as assessed by the Hospital Anxiety and Depression Scale (HADS)), social support (as assessed by the Social Support Questionnaire (SSQ6)) and caregiver burden (as assessed by the Zarit Caregiver Burden Interview) during the first 5 years of their role as caregivers.

Using qualitative approaches, the secondary objectives are: (1) to evaluate the caregiver/patient relationship and changes in this relationship over time as the patient’s disease progresses, (2) to describe the specificities of the attachment relationship between a caregiver and a patient with behavioral disorders (Alzheimer-type dementia in particular) and (3) to study the situations leading to a breakdown in the role of caregiver (nursing home placement, death or disease remission) during the 5 years of participation in the cohort duration.

#### Interventional study

A randomized interventional trial in a 1:1 ratio will be conducted within this cohort to evaluate the effect of a pragmatic supportive intervention on caregivers, provided by a social worker and an information booklet (intervention arm) versus the control arm, where caregivers will only receive the information booklet, without social worker support. The intervention will be conducted during the first 2 years of entrance into the caregiver’s role. The intervention proposed here mobilizes social workers of the Departmental Councils, of the General National Retirement Fund (CARSAT Burgundy-Franche-Comté) and of the Community Social Action Association (CCAS of Dijon). All institutions providing social workers are officially involved in the study and all social workers are trained in the management of older people, characteristics of caregiving relationships and in ICE cohort study procedures. The structure and purposes of the social worker’s intervention are described in Table 1. The main objective is to compare the mental and physical health summary scores on the MOS SF-36 at 1 year between the two groups. Secondary objectives are to compare the following parameters between groups: (1) the mental and physical health summary scores of the MOS SF-36 at 2 years, (2) all HRQoL dimensions of the MOS SF-36 at 1 year and at 2 years, (3) longitudinally: all HRQoL dimensions of the MOS SF-36 and the CarGOQoL questionnaire (only for caregivers of the cancer patient group), (4) coping strategies (as assessed by the coping scale developed by Borteyrou, Truchot and Rascle [35]), (5) anxiety-depression (as assessed by the HADS), (6) social support (as assessed by the SSQ6) and the perceived caregiver burden (as assessed by the Zarit Caregiver Burden Interview) and (7) to assess from a societal perspective the incremental cost-effectiveness ratio (ICER) of the intervention of a social worker for caregivers. The ICER will be expressed in terms of quality-adjusted life years (QALYs) gained based on a utility score that will be calculated based on the EuroQoL EQ-5D questionnaire, the SF-6D which is composed of 11 items of the SF-36 questionnaire and the Center for Epidemiologic Studies-Depression Scale (CES-D) questionnaires and economic survey.

**Table 1:** Content and purposes of the pragmatic intervention by a social worker in the intervention group of the nested randomized trial for caregivers

Format of the intervention:
For each caregiver randomized to the intervention arm of the trial, a social worker will conduct an individual interview with the caregiver
Purposes of the interview:
To simply listen to the caregiver and allow them to express their complaints (and to be attentive to unspoken complaints) To evaluate the level of difficulty experienced by the caregiver in their daily routine, being attentive to their personal situation To respect and respond to any needs and/or requests expressed by the caregiver, and provide active support; guarantee confidentiality of the conversations and ensure that the caregiver participates willingly, without intruding in their life To provide any resources that are lacking in the caregiver-patient relationship; provide information about available solutions for respite care, and other solutions specific to each case (accommodation, health care pathway, logistic/material support for the caregiver/patient) To help the caregiver to more quickly apprehend and come to terms with their commitment of being a caregiver To be attentive to any alert signals and prevent deterioration towards crisis situations (burn-out, abuse); to alert third parties if the caregiver is found to be in a critical situation Through their interactions with the caregiver, the social worker will provide accompaniment and support, offering pertinent information and orientation towards appropriate structures of care depending on the situation the caregiver describes. The intervention leaves it to the discretion of the social worker to decide what is the most appropriate way to operate and the most appropriate solution to propose in practice for each caregiver The intervention also aims to determine whether the needs expressed by the caregivers vary according to the patient’s disease, or according to the patient’s age, sex or place of residence. After the intervention, the social worker will complete a standardized Data Transmission Sheet, with the Linear Analogue Scale Assessment quality of life (LASA) questionnaire (about the caregiver but completed by the social worker), to be forwarded to the study coordinator. They will receive receipt of their submission once the forms have been submitted

The duration of patients’ and caregivers’ participation in the cohort study is 5 years. The entire cohort study’s duration is 10 years: 5 years of inclusions and 5 years of follow-up. Only the interventional study is restricted to 2 years.

The logistic support of the study will be carried out by three clinical research assistants (CRAs) (one in each center) who are involved in the ICE cohort project and who have also participated in the reflection phase during the writing of the study protocol. They will be in charge of patient screening, questionnaire sending, data collection and for following patients/caregivers.

### Study population

#### Inclusion criteria

To be eligible, patients must meet the following inclusion criteria: they must be able to identify a primary caregiver via a specific questionnaire and must consent to complete this (if patients are unable to identify their caregiver themselves due to their disease, a form for self-designation as primary caregiver will be available); patients must be aged 60 years or older and residing in the region of Burgundy-Franche-Comté; they must have recently been diagnosed in hospital or in a private-sector medical practice with one or more of the following diseases: local or metastatic cancer diagnosed within the previous 6 months (breast cancer in first-line chemotherapy treatment, hormone-sensitive prostate cancer, colorectal cancer in first-line chemotherapy treatment); neurodegenerative disease (Parkinson’s disease diagnosed within the previous 5 years, Alzheimer’s or similar diseases diagnosed within the previous 12 months), AMD, (geographic atrophy or neovascular AMD, diagnosed within the previous 12 months, with an acuity range of between 2 and 6/10, aged 65 years or older and able to complete the Mini-Mental State Examination (MMSE) test); heart disease (heart failure diagnosed within the previous 3 months); neurovascular disease (ischemic or hemorrhagic stroke with clinical evidence of post-stroke lesions more than 24 h old, diagnosed within the previous 6 months and with a Rankin score ≤ 2).

*The eligibility criteria of principal caregivers* are as follows: they must be members of the patient’s social environment (family, friend or neighbor); they must be identified by the patient as the “primary caregiver” based on the designation questionnaire or they must have completed the self-designation form as primary caregiver; they must be aged 18 years or older; they must not be employees of a health care organization; they must be residing in the region of Burgundy-Franche-Comté; and they must be able to complete the study questionnaires.

#### Noninclusion criteria

Any patient who has been previously diagnosed with another targeted disease and/or those living in a retirement home will not be included. Principal caregivers who are under guardianship, curatorship or under the protection of justice will be excluded.

#### Inclusion procedure

**Fig. 1:**
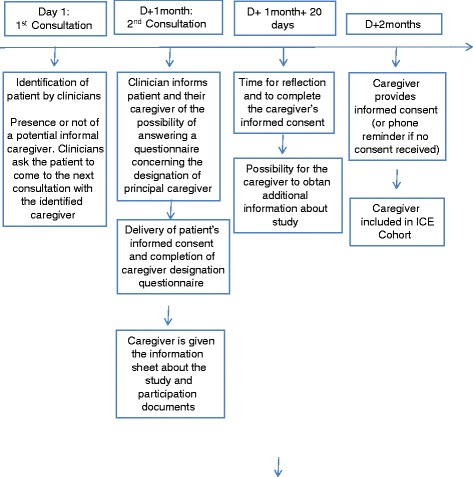
Scenario 1: patients are accompanied by their caregiver. Caregivers’ inclusion according to scenario 1: patients are accompanied by their caregiver

**Fig. 2:**
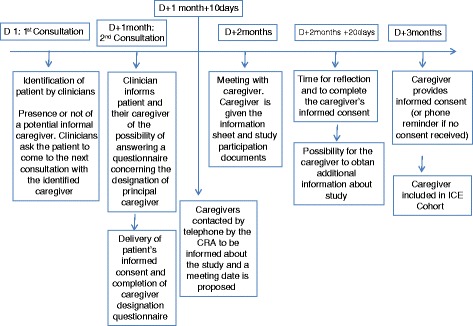
Scenario 2: patients are alone. Caregivers’ inclusion according to scenario 2: patients are alone

Inclusion will be performed in two steps. First, patients will be identified, during a clinical consultation in the context of a diagnosis for one of the diseases being investigated, by clinicians involved in the ICE cohort study who had participated in the reflection phase on inclusion strategies for the different diseases, the procedures to recruit caregivers and the logistic support to set up. At the first consultation, which is a medical visit planned at hospital within the scope of the patient’s pathology, the clinician will indicate the presence or not of a potential informal primary caregiver. They will ask the patient to come to the next consultation with their caregiver (the timetable is defined according to the inclusion strategy, depending on the patient’s pathology). Then, at the second consultation, two possibilities for the inclusion of informal caregivers exist, namely patients are either accompanied by their caregiver or they attend unaccompanied. If the patient comes to the second consultation with their caregiver, as requested, both the patient and their caregiver will be informed of the study, complete the identification questionnaire with the health care facility CRA involved in the study, and receive the Consent Form. Alternatively, if the patient does not attend the consultation with his caregiver, then the caregivers will be contacted by phone based on the information provided by the patient in the identification questionnaire. An appointment will be made with the caregivers to present the study to them and to give them the relevant information concerning the study (information sheet) and the Consent Form. If patients are unable to identify their primary caregiver by themselves due to their health status, a self-designation form as primary caregiver will be proposed to the person(s) accompanying the patient (Figs. 1 and 2).

#### Randomization procedure

Once all the caregivers’ inclusion questionnaires have been completed (during the month following inclusion) and collected by the CRA, randomization will be performed for each group of diseases using a minimisation technique. Minimization will be stratified, regardless of the disease group, according to center, age (80 years or older versus below 80 years) and gender. Additional specific stratification factors are:

1. For cancers: cancer localization (breast, prostate, colorectal), stage (nonmetastatic and resectable versus advanced or metastatic)
2. For Alzheimer’s disease: stage of disease (very early stage (MMSE score ≥ 26) versus mild (20 ≤ MMSE score < 26) versus moderate (10 ≤ MMSE score < 20) versus severe (MMSE score < 10))
3. For Parkinson’s disease: stage of severity of Parkinson’s disease (stages I to V according to the Hoehn and Yahr classification) [19]
4. For heart failure: severity of disease (according to the New York Heart Association (NYHA) classification)
5. For neurovascular diseases: Rankin Score: (nondependent Rankin ≤ 2 or dependent Rankin > 2)
6. For AMD: form of AMD, namely geographic atrophy versus neovascular disease

Caregivers randomized to the intervention arm will receive an information booklet and will receive support provided by a social worker scheduled every 6 months for 2 years (from 6 to 24 months after inclusion). The duration of the support will be 1 h per visit and will be defined in two parts: firstly, administration of the Linear Analogue Scale Assessment quality of life (LASA) questionnaire, and secondly, a standardized semidirective interview to assess needs. The LASA questionnaire will serve as a support tool for the intervention to detect any health problems and optimize the responses provided by the social worker [20]. LASA includes four HRQoL dimensions, namely global quality of life, mental wellbeing, physical wellbeing, and level of fatigue and, using a Visual Analogue Scale (VAS) ranging from 0 (worst HRQoL level imaginable) to 10 (best HRQoL level), assesses the caregiver’s perception of their own health status. The social worker will then conduct a semidirective interview to evaluate the caregiver’s needs and to detect any early signs of burn-out.

In the control arm, caregivers will only receive the information booklet. This booklet will inform caregivers about existing structures and support programs. Nevertheless, caregivers randomized to the control arm retain the possibility to solicit the social services participating in the study on their own initiative, or other appropriate services, for social support if needed.

### Sample size calculation

#### Observational and interventional studies

The calculation of the number of caregivers required was based on both the design and the primary objectives of the intervention study, with noncompetitive recruitment according to each group of diseases (randomization performed 1:1 per group). We aim to demonstrate the efficiency of the intervention in each of the five groups of diseases. The hypothesis is that the intervention proposed will improve the HRQoL of caregivers on at least one of the two summary scores of the MOS SF-36 (i.e., physical health and/or mental health).

The minimal clinically important difference (MCID), which is the smallest change in an outcome that a patient would identify as important, was fixed at 5 points [21].

The null hypothesis (H0) is that there will not be a difference of at least 5 points between the two arms in at least one HRQoL summary score at 1 year; while the alternative hypothesis (H1) is that there will be a difference of at least 5 points between the two arms in at least one HRQoL summary score at 1 year.

With a bilateral alpha risk of 5 % and a statistical power of 90 % to detect a difference of at least 5 points (standard deviation (SD) = 25), and adjusted to take account of the number of statistical tests and maintain an overall alpha type one error rate of 5 % (Bonferroni adjustment):

- With two dimensions of HRQoL, α’ = α/2 = 0.05/2 = 0.025
- With five groups of diseases, α” = α’/5 = 0.025/5 = 0.005 (cancer (breast, prostate, colorectal); neurodegenerative disease (Alzheimer’s disease and similar diseases); AMD; neurovascular (stroke); cardiac (heart failure))
- At least 1684 caregivers should be randomized in each disease group. However, we anticipate lower recruitment in the heart failure group. Accordingly, only 488 caregivers will be randomized (bilateral α type I error of 0.005 and statistical power of 80 %) to demonstrate a difference of at least 5 points (SD = 15). Thus, it will be necessary to include and to randomize 4 × 1684 = 6736 caregivers, plus 488 in the heart failure group, representing a total of 7224 caregivers. An interim analysis will be planned to reject either H0 or H1 (Alpha Spending Function and O’Brien-Fleming Boundaries) when half the caregivers have been included with 1 year of follow-up in each of the subgroups. To allow for 5 % of loss to follow-up, it will be necessary to include and randomize an additional 380 caregivers, resulting in an overall total of 7604 caregivers (1750 caregivers for each of the four large groups of diseases and 604 for the heart failure group)

### Study procedures

#### Data collection

Caregivers and patients’ sociodemographic data (sex, date of birth, place of residence, matrimonial status, study level, professional status) and patients’ medical data (type of disease, stage of disease, treatment) will be collected by the CRA by interviews or from medical files and recorded via CleanWeb Designer™ software version 164.3.0 (Telemedicine Technologies SA).

#### Administration of questionnaires

Caregivers will receive the questionnaires by post or by an email inviting them to complete the questionnaires online via CleanWeb Designer™ software. Each participating caregiver will be free to choose their preferred method for completing the questionnaires. All questionnaires are self-administered. The CRA will carry out sending and receipt of questionnaires. If filled questionnaires are not received 4 weeks after sending, a follow-up call will be performed by the CRA.

**Fig. 3:**
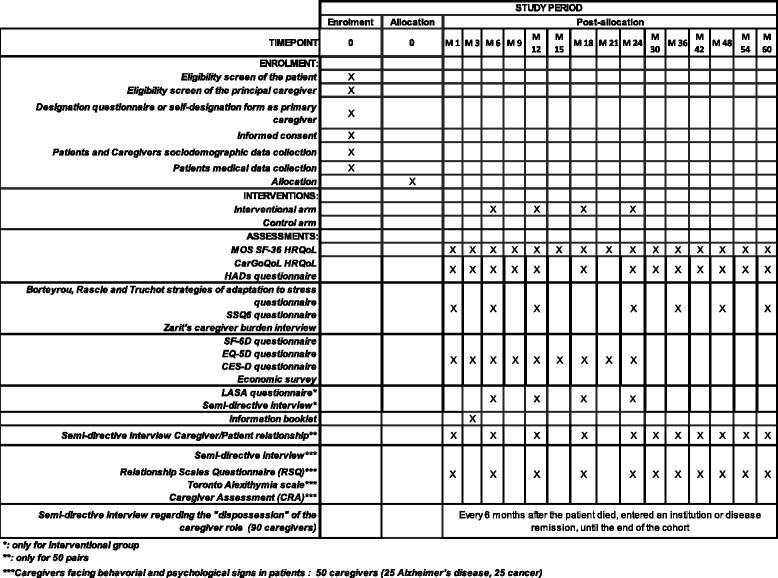
A schematic diagram of time schedule for enrollment, interventions, assessments and visits for participants

A schematic diagram of time schedule for the administration of the different questionnaires is presented in Fig. 3.

#### Health-related Quality of life

The primary endpoint of the study is HRQoL. This will be evaluated using the Medical Outcomes Study (MOS) Short Form (SF)-36, which is a self-administered questionnaire [22–28]. It comprises 36 items divided into eight scales, namely: physical functioning (10 items), role limitations due to physical health (four items), pain (two items), general health perceptions (five items), energy/fatigue (four items), social functioning (two items), role limitations due to emotional problems (three items), mental health (five items) and health change (one item). The average time required for completion is 5 to 10 min. It is the most frequently used generic instrument, and has been translated and validated in the French language in a wide range of diseases [29, 30]. One score is generated per dimension, and normalized on a scale of 0 (worst HRQoL) to 100 (best HRQoL). Physical and mental health summary scores are also generated.

HRQoL will also be evaluated for caregivers of cancer patients using the CareGoQoL questionnaire. This self-administered questionnaire includes 29 items assessing 10 HRQoL dimensions, namely: psychological wellbeing, burden, relationship with health care professionals, financial and administrative management, coping, physical wellbeing, self-esteem, leisure activities, social support and private life. For each individual, scores for a dimension are computed if at least half of its contributive items are answered. The score of each dimension is obtained by computing the mean of the item scores for that dimension. An index is computed as the mean of the dimension scores. All dimension scores and the index are linearly transformed and standardized on a scale of 0–100 (the higher the score, the better the HRQoL). This instrument has recently been validated in French [31].

#### Anxiety and depression

Anxiety and depression will be assessed using the self-administered Hospital Anxiety and Depression Scale (HADS) questionnaire. This includes 14 items distributed in two subscales, namely an anxiety and a depression subscale. Each item is measured on a 0 to 3 scale. The HADS has been used in various medical contexts (hospitalized and nonhospitalized patients as well as apparently healthy persons). It has also been validated in the French language [32–34].

#### Coping

The coping strategies used by caregivers for stress adaptation will be identified using the coping scale developed by Borteyrou, Truchot and Rascle [35]. It includes 25 items to assess six dimensions: escape strategies (behavioral avoidance strategies that allow caregivers to leave the care context), psychological and behavioral disengagement strategies (avoidance strategies to protect against sources of stress), positive reinterpretation strategies (cognitive strategies to distance oneself emotionally from problems encountered), distraction activities or behavioral strategies that aim to restore resources (protective strategies to leave the care context during which caregivers play an active role in entertaining or social activities), and consultations with a psychologist and seeking social support (emotional strategies that aim to verbalize problems, express negative emotions, exteriorize feelings without any obvious desire to find solutions to problems). The validity of this tool has been tested in professional caregivers and noncaregivers in oncology. This coping scale will identify the processes by which caregivers confront the stresses linked to their care relationship.

#### Social support

The six-item Short Form of the SSQ6, which is validated in French, will be used to qualitatively and quantitatively evaluate the caregivers’ social network [36, 37]. The SSQ6 is administered in two steps. During the first step, respondents indicate the name or the initials of people providing each social support described. In this way, a list of persons in the network is established. In the second step, respondents indicate, for each person, their degree of satisfaction with regard to the support received. It is possible to calculate a total score for availability *N* by adding the number of persons available for each type of support. A satisfaction score for the support provided by the network is calculated by adding scores obtained, which vary from 1 to 5 [38].

#### Caregiver burden

The Zarit Caregiver Burden Interview will be used to evaluate the burden represented by caring for a patient living at home [39]. It reveals the degree of exhaustion or psychological fatigue of caregivers. It is composed of 22 items, scored 0 to 4, divided into three dimensions: the relationship with the person being cared for, repercussions on caregivers’ life and their own emotional state. Responses are distributed on a scale of 5 points. The overall score shows whether there is a slight, moderate or heavy burden or no burden at all. In addition to evaluating the material and emotional burden, this inventory also contains a complementary questionnaire of 27 items, scored 0 to 4, centered on the evaluation of problems with the behavior and autonomy of the cared person, scored according to their frequency and the caregivers’ emotional reactions. The score sheet has two parts: one part “frequency” and one part “relationship.” The Zarit Caregiver Burden Interview is a reliable tool that has been validated in French for the assessment of caregiver burden [37].

#### Efficiency

A cost-utility analysis will be performed to compare the two arms of the interventional study (intervention of a social worker for caregivers versus no intervention) in terms of costs and quality-adjusted life years (QALYs). The point of view adopted will be the societal perspective. The societal perspective consists in aggregating all expenditure, regardless of its nature and source of funding (social security, private health insurance, out-of-pocket expenses) [40]. The choice of this perspective is justified by the diversity of the expected consequences of the intervention of a social worker. Only direct medical costs will be taken into account. They will include drugs and hospitalizations, medical consultations, all biological tests, medical/technical procedures, and medical transportation related to the caregivers’ health state. Social security benefits received by caregivers will also be taken into consideration, as well as costs associated with caregiving time and out-of pocket expenses. Productivity costs will not be estimated because it is usually considered that they are included in the QALY estimation. However, the impact of the informal caregiving on the caregivers’ professional situation will be evaluated using the economic survey. QALYs will be calculated based on the use of utility score from the EQ-5D, a multiattribute generic questionnaire composed of six attributes: mobility, self-care, usual activity, pain/discomfort and anxiety/depression. Each attribute has three levels, thus defining 243 possible health states [41, 42]. In a sensitivity analysis, another questionnaire aimed at estimating utility scores will be used, namely the SF-6D. It is composed of 11 items of the SF-36 questionnaire [43, 44]. The Center for Epidemiologic Studies-Depression Scale questionnaire (Center for Epidemiologic Studies-Depression Scale questionnaire (CES-D) will also be used to assess the overall health of caregivers. It is a self-report instrument, composed of 20 items, and validated in French for evaluating depression [45]. Questionnaires will be administrated to caregivers every 3 months during the first 2 years of follow-up.

#### Qualitative studies

The qualitative studies will make it possible to answer the secondary objectives of this research:

1. To study the caregiver-patient relationship, we will conduct semistructured interviews with 50 caregiver-patient pairs (10 pairs in each pathology group). Interviews will be recorded and transcribed in their entirety. These interviews (between 1 and 1.5 h long) will be performed with caregivers and patients separately at baseline and every 6 months thereafter over 5 years. Analysis of the interviews will include: thematic content analysis of each interview (first level of analysis), cross-sectional analysis of the content of the caregiver/patient story (second level), cross-analysis of the narratives collected from each group of caregivers and elements of the relevant literature (third level). These analyses will characterize the predetermination of caregiving, explore the experience of helping, retracing its path and analyzing the mutual perception of the aid relationship and the difficulties encountered.
2. To describe the specificities of the relationship when the caregiver has to face behavioral and psychological problems in the patient (Alzheimer-type dementia in particular), we will compare two groups of 25 caregivers. The first group will be caregivers of patients with dementia, and the second group caregivers of patients with cancer. This study is based on semistructured interviews and auto-administered questionnaires. We will meet each person every 6 months during the first 5 years of their role of caregivers to evaluate the following:
  - The quality of the attachment relationship using the Relationship Scales Questionnaire (RSQ) [46]
  - Alexithymia, using the Toronto Alexithymia Scale (TAS) [47]
  - Negative and positive reactions to caregiving using the Caregiver Reaction Assessment [48].
3. To measure the “dispossession” of the caregiver role when the patient is in remission, enters an institution or dies and the repercussions of this event, and to evaluate the consequences for caregivers, we will perform, every 6 month after the event and until the end of the cohort, semidirective interviews with 90 caregivers whose patient had had a disease remission (30 caregivers), entered an institution (30 caregivers) or died (30 caregivers).

#### Analysis

Analyses will be performed using SAS (version 9.4) (SAS Institute Inc., Cary, NC, USA), R (version 3.2.2) [49] and Stata (v13) (StataCorp LP, TX, USA) software. The statistical analysis plan for the primary and secondary objectives will be written before the database is locked.

All analyses will be on a modified intention-to-treat principle, i.e., including all randomized informal carers regardless of the eligibility criteria and intervention received, with MOS SF-36 scores available at baseline and 1 year. Analyses for the interventional study will be done on an intention-to-treat basis. After Bonferroni adjustment, statistical significance will be fixed at the 0.005 level for primary analyses, and at the 0.05 level for the secondary objectives.

An interim analysis is planned when half of the patients will be included to reject or not H0 (efficacy) or H1 (futility); the Alpha Spending Function with O’Brien-Fleming Boundaries will be used to determine the alpha-level significance at the interim and at the final analysis using EAST® software (Cytel Software Corporation, Cambridge, MA, USA) [50]. The corresponding confidence interval will be computed (1 − alpha level).

Sociodemographic and clinical characteristics will be described at inclusion using frequency (percent) for qualitative variables and mean (SD), or median (min–max) for continuous variables. Data will be compared between randomization arms using the Student *t* test or the nonparametric Mann-Whitney test, or analysis of variance (ANOVA) or the Kruskal-Wallis test for continuous variables and the Chi-squared or Fisher’s exact tests for qualitative variables, as appropriate. Scores will be described at each follow-up time point using mean (SD) and median (min–max) in each arm.

After describing missing data and investigating the mechanism of missing data profiles, imputation will be performed using a multiple imputation method by sensitivity analysis after checking normality, and univariate and multivariate ANOVA adjusted for baseline score will be performed to compare the two summary scores of the SF-36 questionnaire at 1 and 2 years.

Multivariate analyses will be performed including at least the stratification criteria for randomization, as well as variables associated with HRQoL at the 10 % level by univariate analysis. HRQoL data will also be longitudinally analyzed using a mixed model of analysis of variance for repeated measures (or pattern mixture models in case of a missing-not-at-random profile) in order to test an arm effect, time effect and an interaction between arm and time, as well as the effect of other relevant parameters (HADS, coping, clinical parameters, etc.). Some random effects on patients will also be introduced into the model in order to reflect individual trends. Burden, coping and HADS will also be modeled as time-dependent covariables in another model. Time to HRQoL score deterioration will be estimated using the definitions of both Bonnetain et al. [51] and Hamidou et al [52]. Item Response Theory, cluster analyses, structural equation modeling as well as classical factorial analyses will be conducted as complementary analyses to define and validate longitudinal profiles of caregivers. All these analyses will be repeated in each subgroup defined by stratification (including cancer localizations) for sensitivity analyses.

#### Efficiency

Outcomes will be expressed as incremental cost-effectiveness ratios (ICERs) (i.e., the difference in overall mean direct medical costs divided by the difference in mean QALYs between the two groups) and cost-effectiveness acceptability curves. The absence of intervention will be considered as the reference strategy. Costs and QALYs will be discounted at a rate of 3 % per annum. The robustness of results will be assessed through a one-way sensitivity analysis based on the use of the SF-6D. A standard nonparametric bootstrap simulation will also be conducted (10,000 replications) to estimate the 95 % confidence interval of the ICER.

In case of missing assessments concerning utility scores, the last observation will be carried forward. Differences in mean utility scores and mean costs between the intervention and control groups will be assessed by the nonpaired *t* test (for variables with a normal distribution) and the nonparametric Mann-Whitney test (for variables with a nonnormal distribution).

## Discussion

The scientific purpose of the ICE cohort study is to define longitudinal profiles of caregivers and to evaluate pragmatic action to support them. This study should make it possible to improve our knowledge concerning the life course of caregivers (by identifying profiles of the caregivers and understanding the mechanisms involved in changing this profile), to identify their needs and their expectations (in terms of professional support, financial assistance, information and advice to assist in caregiving) and to envisage optimized support for patients with chronic disease who live at home.

The consideration of patients’ social environment is essential in an integrative approach to the management of their disease. In this respect, the difficulties encountered by caregivers may have direct implications on the quality of the care they provide. This study will allow us to identify factors associated with modifications in patient management support. Improved knowledge of the difficulties that caregivers face in their everyday lives could contribute to identifying potentially high-risk situations in the care relationship (e.g., mistreatment) and make it possible to implement timely support for caregivers at risk of psychological, physical or social distress.

The purpose, in terms of public health, is to avoid the health deterioration of caregivers, and thus indirectly, to improve the patient’s health status through optimized management that is potentially inexpensive and economically acceptable for society. The intervention study is based on the hypothesis that early intervention during the initial phase of the care relationship is likely to improve the caregivers’ HRQoL and the quality of the support provided. The hypothesis of the associated efficacy study is that the intervention of a social worker, through their role in listening, alerting, advising and guiding, may improve the wellbeing of informal caregivers, and thus reduce their potential consumption of health care resources, and consequently, the expenditure related to their care mission. Improving caregivers’ HRQoL and preventing degradation of their health will make it possible to positively influence the patients’ HRQoL, and as a result, their health, autonomy and capacity to continue living at home. To ensure the homogeneity of the intervention across the Burgundy-Franche-Comté region, all registered social workers in the region will undergo study-specific training. The intervention to be provided in the context of this study by social workers is explained in detail and formalized in a partnership agreement signed by all the social services employing social workers in the region.

The ICE cohort may lead to the formulation of health recommendations and serve as a basis for the development of services that offer support to caregivers to reduce the potential risk of social inequalities related to caregiving.

## Trial status

At the time of manuscript submission, a feasibility study conducted to test the inclusion mechanisms in the clinical departments, as well as the questionnaire concerning the designation of the principal caregiver, has been completed. The feasibility study that was carried out from 2013 to 2015 allowed us to test the acceptability of the study and the questionnaires with the patients and their caregivers, to better understand the logistic and operational aspects. The project protocol has been modified to reflect the results of the feasibility study. Official recruitment to the ICE cohort started in September 2015. The first patient was recruited on 2 October 2015.

## Abbreviations

AMD: Age-related macular degeneration
ANOVA: Analysis of variance
CarGOQoL: CareGiver Oncology Quality of Life
CES-D: Center for Epidemiologic Studies-Depression Scale questionnaire
CRA: Clinical Research Assistant
EQ-5D: European Quality of life – 5 dimensions questionnaire
HADS: Hospital Anxiety and Depression Scale
HRQoL: Health-related quality of life
ICER: Incremental Cost-Effectiveness Ratio
LASA: Linear Analogue Scale Assessment quality of life questionnaire
MCID: Minimal Clinically Important Ddifference
MMSE: Mini Mental State Examination
MOS SF-36: Medical Outcome Study Short Form-36 items
NYHA: New York Heart Association
QALY: Quality-adjusted life year
RSQ: Relationship Scales Questionnaire
SD: Standard deviation
SF-6D: Short Form – 6 dimensions
SSQ6: Six-item Short Form of the Social Support Questionnaire
TAS: Toronto Alexithymia Scale
VAS: Visual Analogue Scale
